# Hungarian adaptation of the Honest Open Proud program

**DOI:** 10.1192/j.eurpsy.2024.587

**Published:** 2024-08-27

**Authors:** D. Őri, P. W. Corrigan

**Affiliations:** ^1^Institute of Behavioural Sciences, Semmelweis University; ^2^Department of Mental Health, Heim Pal National Pediatric Institute, Budapest, Hungary; ^3^Department of Psychology, Illinois Institute of Technology, Chicago, United States

## Abstract

**Introduction:**

The Honest, Open, Proud (HOP) program is an effective peer-led group program to support people with mental health problems in their disclosure to manage self and public stigma. The HOP program will be integrated into the National Anti-stigma Program in Hungary, which was initiated in 2020.

**Objectives:**

Our goal was to develop the Hungarian version of the HOP program. We conducted the following measures to achieve our aim.

**Methods:**

The adaptation process was conducted using community-based participatory research (CBPR) between September 2022 and January 2023. Over ten sessions, a group of eight individuals, consisting of both males and females with varying mental health conditions (mean age = 39.6 ± 8.5), participated in the online-led CBPR. The adaptation process was systematically documented, and regular supervision was provided.

**Results:**

The program comprises three lessons and a follow-up section. We have translated the text of the manual and workbook into Hungarian and adjusted the tone, language, locations, and examples as per the Hungarian context. Although our adaptation process did not involve changes to the content and implementation strategies, we will perform structural modifications and adjustments to ensure the content is suitable for the predefined number of sessions and Hungarian participants.

**Conclusions:**

The HOP could be feasibly implemented in the National Anti-stigma Program in Hungary; both online and in-person programs are planned. Given the lack of such a program in Hungary, it will likely be warmly welcomed and strongly supported for the benefit of people with mental health problems.

**Disclosure of Interest:**

D. Őri Grant / Research support from: Fulbright Association supported research, P. Corrigan: None Declared

